# 3-(1,3-Dioxolan-2-yl)-2-hydrazino-7-methyl­quinoline

**DOI:** 10.1107/S1600536809003031

**Published:** 2009-01-28

**Authors:** R. Subashini, Venkatesha R. Hathwar, P. Nithya, K. Prabakaran, F. Nawaz Khan

**Affiliations:** a Chemistry Division, School of Science and Humanities, VIT University, Vellore 632 014, Tamil Nadu, India; b Solid State and Structural Chemistry Unit, Indian Institute of Science, Bangalore 560 012, Karnataka, India

## Abstract

In the title mol­ecule, C_13_H_15_N_3_O_2_, the dihedral angle between the mean plane of the 1,3-dioxolane group and the 2-hydrazino-7-methyl­isoquinoline unit is 85.21 (5)°. The conformation of the mol­ecule is influenced by bifurcated N—H⋯(O,O) and N—H⋯N intra­molecular hydrogen bonds. In the crystal structure, mol­ecules are linked *via* inter­molecular N—H⋯O hydrogen bonds, forming extended chains along [001].

## Related literature

For general background to hydrazine compounds, see: Broadhurst *et al*. (2001 ▶); Behrens (1999 ▶); Broadhurst (1991 ▶); Chao *et al.* (1999 ▶); Kametani (1968 ▶). For related crystal structures, see: Yang *et al.* (2008 ▶); Choudhury & Guru Row (2006 ▶); Choudhury *et al.* (2002 ▶); Hathwar *et al.* (2008 ▶); Cho *et al.* (2002 ▶); Manivel *et al.* (2009 ▶), and references therein. For bond-length data, see: Allen *et al.*, 1987 ▶)
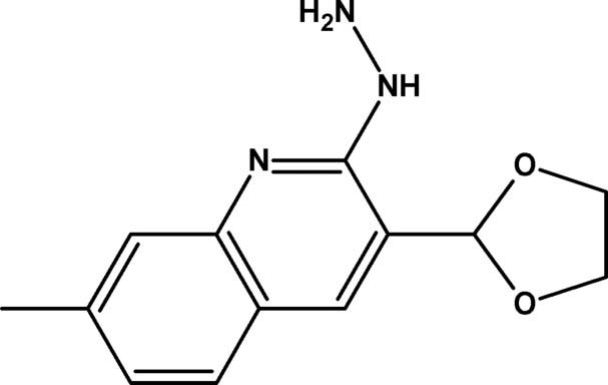


## Experimental

### 

#### Crystal data

C_13_H_15_N_3_O_2_
*M* *_r_* = 245.28
Monoclinic, 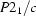

*a* = 13.1909 (17) Å
*b* = 10.1165 (13) Å
*c* = 9.7805 (13) Å
β = 109.956 (2)°
*V* = 1226.8 (3) Å^3^
*Z* = 4
Mo *K*α radiation
μ = 0.09 mm^−1^
*T* = 290 (2) K
0.30 × 0.21 × 0.14 mm

#### Data collection

Bruker SMART CCD area-detector diffractometer
Absorption correction: multi-scan (*SADABS*; Sheldrick, 1996 ▶) *T*_min_ = 0.942, *T*_max_ = 0.987
8929 measured reflections
2279 independent reflections
1699 reflections with *I* > 2σ(*I*)
*R*_int_ = 0.018

#### Refinement

*R*[*F* ^2^ > 2σ(*F* ^2^)] = 0.044
*wR*(*F* ^2^) = 0.129
*S* = 1.06
2279 reflections
176 parameters
H atoms treated by a mixture of independent and constrained refinement
Δρ_max_ = 0.19 e Å^−3^
Δρ_min_ = −0.14 e Å^−3^

### 

Data collection: *SMART* (Bruker, 2004 ▶); cell refinement: *SAINT* (Bruker, 2004 ▶); data reduction: *SAINT*; program(s) used to solve structure: *SHELXTL* (Sheldrick, 2008 ▶); program(s) used to refine structure: *SHELXL97* (Sheldrick, 2008 ▶); molecular graphics: *ORTEP-3* (Farrugia, 1997 ▶) and *PLATON* (Spek, 2003 ▶); software used to prepare material for publication: *PLATON*.

## Supplementary Material

Crystal structure: contains datablocks global, I. DOI: 10.1107/S1600536809003031/lh2748sup1.cif

Structure factors: contains datablocks I. DOI: 10.1107/S1600536809003031/lh2748Isup2.hkl

Additional supplementary materials:  crystallographic information; 3D view; checkCIF report

## Figures and Tables

**Table 1:** Hydrogen-bond geometry (Å, °)

*D*—H⋯*A*	*D*—H	H⋯*A*	*D*⋯*A*	*D*—H⋯*A*
N2—H2N⋯O1	0.843 (19)	2.372 (18)	2.9329 (17)	124.5 (15)
N2—H2N⋯O2	0.843 (19)	2.653 (18)	3.0968 (19)	114.3 (14)
N3—H3N*A*⋯N1	0.94 (2)	2.35 (2)	2.691 (2)	100.9 (15)
N3—H3N*B*⋯O2^i^	0.92 (2)	2.44 (2)	3.207 (2)	141.2 (19)

Symmetry code: (i) 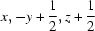.

**Fig. 1.**
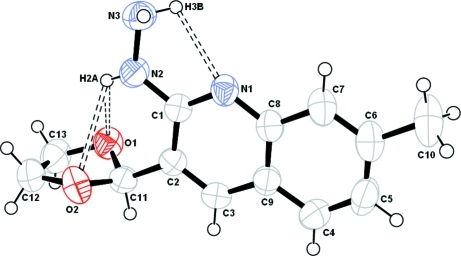
The molecular structure of (I) with 50% probability displacement ellipsoids. Dashed lines indicate hydrogen bonds.

**Fig. 2.**
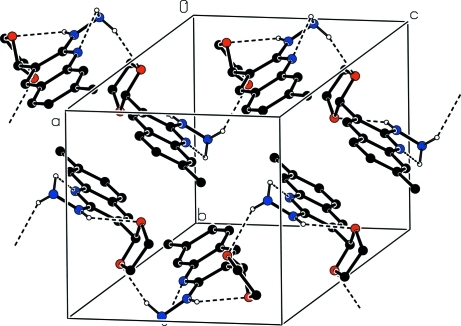
Part of the crystal structure of (I) showing hydrogen bonds as dashed lines. H atoms not involved in hydrogen bonds have been omitted.

## supplementary crystallographic information

### Comment

The title compound (I), belongs to the quinoline class. Quinolines and quinolinones are an integral part of many naturally occurring fused heterocycles and find application in synthetic and pharmaceutical chemistry (Kametani, 1968). Isoquinolinones and isoquinolineamines have been reported as cancer chemotherapeutic agents (Behrens, 1999) whereas quinolyl and isoquinolyl derivatives have been reported as insecticidal compounds (Broadhurst, 1991). 3-substituted isoquinolines have potent use in medicine (Chao *et al.*, 1999) and in general, hydrazine derivatives can be used as medicaments (Broadhurst *et al.*, 2001; Choudhury, *et al.*, 2002; Choudhury & Guru Row, 2006; Yang, *et al.*, 2008). Due to the importance of quinoline derivates (Cho *et al.*, 2002) and in continuous of our research on quinolines and isoquinoline derivatives (Hathwar *et al.*, 2008; Manivel *et al.*, 2009) we present here crystal structure of the title compound.

In (I) the dihedral angle between 1,3-dioxolane moiety and 2 hyrazino-7-methyl isoquinoline unit is 85.21 (5)°. All bond lengths (Allen *et al.*, 1987) and angles are within normal ranges. The conformation of the molecule is influenced by N—H···O and N—H···N intramolecular hydrogen bonds whereas the crystal structure is stabilized by intermolecular N—H···O hydrogen bonds forming exteded chains along [001].

### Experimental

A solution of 2-chloro (3-(1,3-dioxolan-2-yl)-7-methylquinoline in ethanol was treated with hydrazine hydrate and stirred at 323 K for 3hr. The product was filtered. The solid was washed with water and diethyl ether and dried under vacuum. Single crystals were obtained by recrystalization of (I) from DMSO.

### Refinement

All H atoms positioned geometrically and refined using a riding model with bond lengths C—H = 0.93 Å (for aromatic), 0.97 Å (for methylene) and 0.96 Å (for methyl). The *U*_iso_(H) = 1.5*U*_eq_(C) for methyl and *U*_iso_(H) = 1.2*U*_eq_(C) for all other carbon bound H atoms. H atoms bonded to N atoms were located in difference Fourier maps and refined isotropically.

### Crystal data

**Table:**

C_13_H_15_N_3_O_2_	*F*(000) = 520
*M**_r_* = 245.28	*D*_x_ = 1.328 Mg m^−^^3^
Monoclinic, *P*2_1_/*c*	Mo *K*α radiation, λ = 0.71073 Å
Hall symbol: -P 2ybc	Cell parameters from 948 reflections
*a* = 13.1909 (17) Å	θ = 1.8–24.6°
*b* = 10.1165 (13) Å	µ = 0.09 mm^−^^1^
*c* = 9.7805 (13) Å	*T* = 290 K
β = 109.956 (2)°	Block, brown
*V* = 1226.8 (3) Å^3^	0.30 × 0.21 × 0.14 mm
*Z* = 4	

### Data collection

**Table:**

Bruker SMART CCD area-detector diffractometer	2279 independent reflections
Radiation source: fine-focus sealed tube	1699 reflections with *I* > 2σ(*I*)
graphite	*R*_int_ = 0.018
φ and ω scans	θ_max_ = 25.5°, θ_min_ = 1.6°
Absorption correction: multi-scan (*SADABS*; Sheldrick, 1996)	*h* = −15→15
*T*_min_ = 0.942, *T*_max_ = 0.987	*k* = −10→12
8929 measured reflections	*l* = −11→11

### Refinement

**Table:**

Refinement on *F*^2^	Primary atom site location: structure-invariant direct methods
Least-squares matrix: full	Secondary atom site location: difference Fourier map
*R*[*F*^2^ > 2σ(*F*^2^)] = 0.044	Hydrogen site location: inferred from neighbouring sites
*wR*(*F*^2^) = 0.129	H atoms treated by a mixture of independent and constrained refinement
*S* = 1.06	*w* = 1/[σ^2^(*F*_o_^2^) + (0.072*P*)^2^ + 0.1104*P*] where *P* = (*F*_o_^2^ + 2*F*_c_^2^)/3
2279 reflections	(Δ/σ)_max_ < 0.001
176 parameters	Δρ_max_ = 0.19 e Å^−^^3^
0 restraints	Δρ_min_ = −0.14 e Å^−^^3^

### Special details

**Table:**

Geometry. All esds (except the esd in the dihedral angle between two l.s. planes) are estimated using the full covariance matrix. The cell esds are taken into account individually in the estimation of esds in distances, angles and torsion angles; correlations between esds in cell parameters are only used when they are defined by crystal symmetry. An approximate (isotropic) treatment of cell esds is used for estimating esds involving l.s. planes.
Refinement. Refinement of *F*^2^ against ALL reflections. The weighted *R*-factor *wR* and goodness of fit *S* are based on *F*^2^, conventional *R*-factors *R* are based on *F*, with *F* set to zero for negative *F*^2^. The threshold expression of *F*^2^ > σ(*F*^2^) is used only for calculating *R*-factors(gt) *etc*. and is not relevant to the choice of reflections for refinement. *R*-factors based on *F*^2^ are statistically about twice as large as those based on *F*, and *R*- factors based on ALL data will be even larger.

### Fractional atomic coordinates and isotropic or equivalent isotropic displacement parameters (Å^2^)

**Table:**

	*x*	*y*	*z*	*U*_iso_*/*U*_eq_	
O1	0.10652 (9)	0.42297 (12)	−0.28334 (11)	0.0621 (4)	
O2	0.09919 (10)	0.22186 (13)	−0.18872 (12)	0.0701 (4)	
N1	0.32415 (10)	0.45872 (13)	0.15262 (13)	0.0524 (4)	
N2	0.14264 (11)	0.45599 (16)	0.02767 (15)	0.0597 (4)	
N3	0.12501 (13)	0.53613 (19)	0.13548 (18)	0.0664 (4)	
C1	0.24495 (12)	0.42351 (15)	0.03448 (15)	0.0464 (4)	
C2	0.26133 (12)	0.35152 (15)	−0.08326 (15)	0.0480 (4)	
C3	0.36404 (13)	0.31857 (15)	−0.06896 (17)	0.0550 (4)	
H3A	0.3772	0.2726	−0.1435	0.066*	
C4	0.56033 (15)	0.32091 (18)	0.0793 (2)	0.0688 (5)	
H4A	0.5778	0.2750	0.0080	0.083*	
C5	0.64009 (14)	0.35734 (19)	0.2051 (2)	0.0732 (6)	
H5A	0.7111	0.3344	0.2186	0.088*	
C6	0.61719 (14)	0.4286 (2)	0.3145 (2)	0.0662 (5)	
C7	0.51244 (13)	0.46184 (19)	0.29207 (17)	0.0615 (5)	
H7A	0.4966	0.5107	0.3628	0.074*	
C8	0.42773 (12)	0.42464 (15)	0.16556 (16)	0.0496 (4)	
C9	0.45173 (12)	0.35270 (15)	0.05728 (17)	0.0533 (4)	
C10	0.70689 (16)	0.4661 (3)	0.4525 (2)	0.0949 (8)	
H10A	0.6767	0.4936	0.5244	0.142*	
H10B	0.7481	0.5372	0.4329	0.142*	
H10C	0.7530	0.3911	0.4882	0.142*	
C11	0.17109 (13)	0.31376 (16)	−0.21833 (17)	0.0540 (4)	
H11A	0.2010	0.2745	−0.2879	0.065*	
C12	−0.00343 (14)	0.2421 (2)	−0.2979 (2)	0.0782 (6)	
H12A	−0.0588	0.2540	−0.2544	0.094*	
H12B	−0.0228	0.1675	−0.3640	0.094*	
C13	0.00918 (15)	0.3653 (2)	−0.37670 (19)	0.0764 (6)	
H13A	0.0146	0.3440	−0.4706	0.092*	
H13B	−0.0513	0.4247	−0.3910	0.092*	
H2N	0.0904 (15)	0.4447 (17)	−0.050 (2)	0.064 (5)*	
H3NB	0.1453 (18)	0.484 (2)	0.218 (2)	0.095 (7)*	
H3NA	0.1821 (17)	0.597 (2)	0.158 (2)	0.078 (6)*	

### Atomic displacement parameters (Å^2^)

**Table:**

	*U*^11^	*U*^22^	*U*^33^	*U*^12^	*U*^13^	*U*^23^
O1	0.0577 (7)	0.0724 (8)	0.0494 (6)	0.0026 (6)	0.0095 (5)	0.0034 (5)
O2	0.0667 (8)	0.0722 (8)	0.0642 (7)	−0.0201 (6)	0.0130 (6)	−0.0040 (6)
N1	0.0475 (8)	0.0647 (9)	0.0438 (7)	−0.0047 (6)	0.0141 (6)	−0.0015 (6)
N2	0.0476 (8)	0.0838 (11)	0.0443 (7)	0.0051 (7)	0.0114 (6)	−0.0113 (7)
N3	0.0626 (10)	0.0782 (11)	0.0586 (9)	0.0087 (9)	0.0211 (7)	−0.0131 (9)
C1	0.0467 (8)	0.0495 (9)	0.0426 (8)	0.0001 (7)	0.0145 (7)	0.0037 (6)
C2	0.0497 (9)	0.0465 (8)	0.0470 (8)	0.0006 (7)	0.0153 (7)	0.0012 (6)
C3	0.0580 (10)	0.0492 (9)	0.0589 (9)	0.0019 (7)	0.0214 (8)	−0.0077 (7)
C4	0.0566 (10)	0.0607 (11)	0.0890 (13)	0.0055 (8)	0.0245 (9)	−0.0053 (10)
C5	0.0424 (9)	0.0707 (12)	0.0974 (14)	0.0022 (8)	0.0122 (9)	0.0105 (11)
C6	0.0518 (10)	0.0774 (13)	0.0628 (11)	−0.0136 (9)	0.0108 (8)	0.0122 (9)
C7	0.0526 (10)	0.0785 (12)	0.0512 (9)	−0.0134 (8)	0.0148 (8)	0.0024 (8)
C8	0.0480 (9)	0.0526 (9)	0.0470 (8)	−0.0057 (7)	0.0147 (7)	0.0065 (7)
C9	0.0474 (9)	0.0470 (9)	0.0629 (10)	0.0001 (7)	0.0156 (7)	0.0035 (7)
C10	0.0550 (11)	0.138 (2)	0.0771 (13)	−0.0265 (12)	0.0040 (10)	0.0078 (13)
C11	0.0531 (9)	0.0597 (10)	0.0492 (9)	−0.0007 (7)	0.0175 (7)	−0.0083 (7)
C12	0.0554 (11)	0.0901 (15)	0.0852 (13)	−0.0112 (10)	0.0188 (10)	−0.0274 (12)
C13	0.0565 (11)	0.1135 (17)	0.0500 (9)	0.0012 (11)	0.0065 (8)	−0.0119 (11)

### Geometric parameters (Å, °)

**Table:**

O1—C11	1.4074 (19)	C4—H4A	0.9300
O1—C13	1.422 (2)	C5—C6	1.406 (3)
O2—C12	1.424 (2)	C5—H5A	0.9300
O2—C11	1.427 (2)	C6—C7	1.365 (3)
N1—C1	1.3151 (18)	C6—C10	1.509 (2)
N1—C8	1.372 (2)	C7—C8	1.406 (2)
N2—C1	1.3683 (19)	C7—H7A	0.9300
N2—N3	1.411 (2)	C8—C9	1.407 (2)
N2—H2N	0.843 (19)	C10—H10A	0.9600
N3—H3NB	0.92 (2)	C10—H10B	0.9600
N3—H3NA	0.94 (2)	C10—H10C	0.9600
C1—C2	1.440 (2)	C11—H11A	0.9800
C2—C3	1.355 (2)	C12—C13	1.504 (3)
C2—C11	1.495 (2)	C12—H12A	0.9700
C3—C9	1.417 (2)	C12—H12B	0.9700
C3—H3A	0.9300	C13—H13A	0.9700
C4—C5	1.368 (3)	C13—H13B	0.9700
C4—C9	1.411 (2)		
			
C11—O1—C13	104.05 (14)	N1—C8—C7	118.75 (15)
C12—O2—C11	106.35 (14)	N1—C8—C9	122.22 (14)
C1—N1—C8	118.70 (13)	C7—C8—C9	119.03 (15)
C1—N2—N3	120.87 (13)	C8—C9—C4	118.70 (15)
C1—N2—H2N	120.1 (12)	C8—C9—C3	117.09 (14)
N3—N2—H2N	117.4 (12)	C4—C9—C3	124.20 (16)
N2—N3—H3NB	104.8 (14)	C6—C10—H10A	109.5
N2—N3—H3NA	102.9 (12)	C6—C10—H10B	109.5
H3NB—N3—H3NA	101.6 (18)	H10A—C10—H10B	109.5
N1—C1—N2	116.89 (14)	C6—C10—H10C	109.5
N1—C1—C2	123.28 (14)	H10A—C10—H10C	109.5
N2—C1—C2	119.82 (13)	H10B—C10—H10C	109.5
C3—C2—C1	117.35 (13)	O1—C11—O2	105.16 (13)
C3—C2—C11	119.66 (14)	O1—C11—C2	112.04 (13)
C1—C2—C11	122.99 (13)	O2—C11—C2	111.79 (13)
C2—C3—C9	121.34 (15)	O1—C11—H11A	109.2
C2—C3—H3A	119.3	O2—C11—H11A	109.2
C9—C3—H3A	119.3	C2—C11—H11A	109.2
C5—C4—C9	120.33 (18)	O2—C12—C13	105.10 (14)
C5—C4—H4A	119.8	O2—C12—H12A	110.7
C9—C4—H4A	119.8	C13—C12—H12A	110.7
C4—C5—C6	121.55 (17)	O2—C12—H12B	110.7
C4—C5—H5A	119.2	C13—C12—H12B	110.7
C6—C5—H5A	119.2	H12A—C12—H12B	108.8
C7—C6—C5	118.21 (16)	O1—C13—C12	104.14 (14)
C7—C6—C10	121.57 (19)	O1—C13—H13A	110.9
C5—C6—C10	120.22 (17)	C12—C13—H13A	110.9
C6—C7—C8	122.15 (17)	O1—C13—H13B	110.9
C6—C7—H7A	118.9	C12—C13—H13B	110.9
C8—C7—H7A	118.9	H13A—C13—H13B	108.9

### Hydrogen-bond geometry (Å, °)

**Table:**

*D*—H···*A*	*D*—H	H···*A*	*D*···*A*	*D*—H···*A*
N2—H2N···O1	0.843 (19)	2.372 (18)	2.9329 (17)	124.5 (15)
N2—H2N···O2	0.843 (19)	2.653 (18)	3.0968 (19)	114.3 (14)
N3—H3NA···N1	0.94 (2)	2.35 (2)	2.691 (2)	100.9 (15)
N3—H3NB···O2^i^	0.92 (2)	2.44 (2)	3.207 (2)	141.2 (19)

Symmetry codes: (i) *x*, −*y*+1/2, *z*+1/2.
